# Incidence of Proteinuria Post Radical Nephrectomy in Comparison to Partial Nephrectomy: A Comparative Study

**DOI:** 10.7759/cureus.76548

**Published:** 2024-12-28

**Authors:** Ahmed Alasker, Rakan A Al Muammar, Abdulrahman A Bin Moammar, Hassan Alqahtani, Abdulrahman S Altowaim

**Affiliations:** 1 Department of Urology, National Guard Hospital, Riyadh, SAU; 2 College of Medicine, King Saud Bin Abdulaziz University for Health Sciences, Riyadh, SAU

**Keywords:** mortality, nephrectomy, partial nephrectomy, proteinuria, radical nephrectomy

## Abstract

Objectives

The objective of this study is to enhance understanding of the incidence and impact of proteinuria following nephrectomy, to guide clinical decision-making, and to optimize post-operative monitoring strategies. Specifically, the study seeks to compare the incidence of proteinuria in patients undergoing radical nephrectomy and those receiving partial nephrectomy, thereby contributing valuable insights into post-surgical outcomes that could inform treatment approaches and improve patient care.

Methods

It is a retrospective cohort design, analyzing clinical data from patients who underwent radical or partial nephrectomy in King Abdulaziz Medical City (KAMC), Riyadh, Saudi Arabia, between 2014 and 2022. Data was entered in Excel (Microsoft Corporation, Redmond, Washington, United States) and analyzed in IBM SPSS Statistics for Windows, Version 29.0 (Released 2023; IBM Corp., Armonk, New York, United States).

Results

There was a total of 310 participants, predominantly male (n=167, 53.9%), with radical nephrectomy (n=188, 60.6%) being more common than partial (n=99, 31.9%). Post surgery, a significant decline in estimated glomerular filtration rate (eGFR) was noted in radical nephrectomy at one to three months (73.09 mL/minute) compared to partial nephrectomy (90.99 mL/minute) (p<0.001), with similar trends at 6-12 months. The mortality rate was low at 1.6% (n=5), with significant associations between preoperative eGFR and mortality (p=0.008). Proteinuria post operation was observed in 27.1% (n=84), with significant differences in proteinuria levels between radical (107.10 mg/dL) and partial nephrectomy (62.80 mg/dL) (p=0.031).

Conclusion

Our study found that radical nephrectomy was more common and associated with a greater decline in postoperative eGFR compared to partial nephrectomy. Proteinuria was significantly higher in radical nephrectomy patients, and preoperative eGFR was linked to mortality risk, highlighting the need for careful monitoring in high-risk individuals.

## Introduction

Nephrectomy is a known operation used to treat kidney cancer and benign conditions and for renal transplant [1]. In general, there are two types of nephrectomies: partial and radical [2]. Partial nephrectomy is the removal of part of the kidney or tumor preserving the rest of the kidney and it is used for localized or slightly small tumors <4 cm [3]. On the other hand, radical nephrectomy is the removal of the entire kidney, ureter, and adrenal gland [4]. Both operations can be done as laparoscopic, robotically assisted, or open surgery [2].

Several studies have investigated the incidence of proteinuria following nephrectomy, but a direct comparison between radical nephrectomy and partial nephrectomy is lacking. One study examined the occurrence of pathological proteinuria in remnant kidneys after unilateral nephrectomy [5]. Their findings revealed a high incidence of pathological proteinuria, with 82.1% of remnant kidneys exhibiting proteinuria, suggesting an increased risk of proteinuria and kidney diseases following unilateral nephrectomy [5]. The study provides evidence supporting the notion that unilateral nephrectomy may have detrimental effects on kidney function.

A more recent study by Sun et al. investigated the relationship between the urine albumin-to-creatinine ratio and kidney function after nephrectomy [6]. Their study included 1,930 patients who underwent either radical or partial nephrectomy. The results showed that albuminuria severity, as indicated by the urine albumin-to-creatinine ratio, was independently associated with progressive chronic kidney disease (CKD) after both radical and partial nephrectomy. This suggests that the severity of albuminuria can be a valuable marker for assessing kidney function and predicting long-term outcomes following nephrectomy [6]. Another study examined kidney function post-nephrectomy due to various etiologies, emphasizing differences in CKD development between donor nephrectomy and other nephrectomy causes, indicating a higher risk in diseased kidneys compared to donor kidneys [7].

Groen In 't Woud et al. conducted a meta-analysis on childhood unilateral nephrectomy cases, finding a notable prevalence of kidney injury post-surgery, regardless of the indication for nephrectomy [8]. Praga et al.'s study investigated the influence of obesity on proteinuria and renal insufficiency post-unilateral nephrectomy, revealing a higher risk in obese patients for developing proteinuria and renal insufficiency over time [9]. A study by Lee et al. identified risk factors for proteinuria development after radical nephrectomy for renal cell carcinoma (RCC), highlighting compensatory adaptations such as structural hypertrophy and functional hyperfiltration that reduce the occurrence of proteinuria in some patients [10].

While these studies provide useful insights into proteinuria outcomes following nephrectomy, a comparative study focusing on the incidence of proteinuria after radical nephrectomy versus partial nephrectomy would provide a more comprehensive understanding of the differences between these surgical approaches and their effects on proteinuria development.

The purpose of this study is to fill up important knowledge gaps regarding the incidence of proteinuria in post-radical nephrectomy in comparison to partial nephrectomy for better understanding when weighing the benefits and risks in both surgical procedures. It will concentrate on offering specific data between 2014 and 2022 in King Abdulaziz Medical City (KAMC), Riyadh, Saudi Arabia. Also, the goals are to analyze clinical data and assess the severity of the risk factors associated with radical and partial nephrectomy. This understanding could have implications for improving patient care and healthcare policies in Saudi Arabia.

## Materials and methods

This was a retrospective cohort study conducted in the Department of Urology, KAMC, Riyadh, Saudi Arabia, between 2014 and 2022. KAMC was founded in May 1983 and since then it continued expanding the range of medical services it provides. The study was approved by King Abdullah International Medical Research Center (approval number: 0000034024).

Inclusion and exclusion criteria

The inclusion criteria for this study included patients who had undergone either partial or radical nephrectomy with complete medical records. The study also considered patients with relevant risk factors, such as hypertension (HTN), diabetes mellitus (DM), and lifestyle factors. Both male and female patients aged 15 years and older were eligible for inclusion.Exclusion criteria for this study include patients with incomplete electronic medical records or missing essential variables, as well as those with a diagnosis of end-stage renal disease before undergoing nephrectomy.

Sampling approach

A consecutive non-probability sampling technique was employed to obtain eligible patient records, ensuring the inclusion of every patient meeting the defined inclusion and exclusion criteria within the study period. Given the qualitative and exploratory nature of the study, a precise statistical sample size calculation is not applicable, as the sampling approach is not intended to achieve probabilistic representativeness of the population.

Data collection and definitions

All necessary data and relevant information were retrieved from the BestCare medical records system at KAMC in Riyadh, Saudi Arabia. This approach facilitated comprehensive data collection across all eligible cases without aiming for probabilistic representativeness. Data collection included variables related to patient demographics, surgical details, and postoperative outcomes, such as age, body mass index (BMI), nephrectomy type (radical or partial), and pre- and post-surgery serum creatinine levels. Proteinuria was measured using standardized laboratory methods, quantified, and documented at designated intervals (pre- and post-operation).

Case definitions for proteinuria were established following accepted clinical guidelines, categorizing proteinuria levels as normal, mild, moderate, or severe.

Data analysis

Data was entered in Excel (Microsoft Corporation, Redmond, Washington, United States). Data analysis was performed to compare the incidence of proteinuria following radical versus partial nephrectomy. Descriptive statistics summarized participant demographics and clinical characteristics, with means and standard deviations (SDs) calculated for continuous variables and frequencies for categorical variables. The incidence of proteinuria between the two nephrectomy types was compared using Chi-square tests for categorical outcomes and independent t-tests for continuous variables. To evaluate the effect of nephrectomy type on the likelihood of developing proteinuria, a logistic regression model was used, adjusting for confounders such as age, gender, and preoperative kidney function.

Comprehensive statistical analyses were conducted on the dataset, utilizing both descriptive and inferential methods. Descriptive analysis summarized demographic characteristics, including age, gender, and other relevant factors. For categorical variable associations, Chi-square tests and Fisher’s Exact Test were used, while differences between continuous variables were assessed with independent t-tests. All statistical analyses were conducted using IBM SPSS Statistics for Windows, Version 29.0 (Released 2023; IBM Corp., Armonk, New York, United States), with a p-value of less than 0.05 considered statistically significant.

## Results

The study included 310 participants, predominantly male (n=167, 53.9%) with female participants accounting for 143 (46.1%) (Table 1). The average age of participants was 53.8 years (SD ±15.5), ranging from 19 to 92 years. The types of nephrectomies performed were mostly radical (n=188 (60.6%), followed by partial (n=99, 31.9%), with a few cases of nephroureterectomy (n=15, 4.8%) and simple/total nephrectomy (n=7, 2.3%). Laparoscopy was the most common procedure (n=172, 55.5%), with open and robotic procedures each accounting for 22.3% (n=69). The primary indication for surgery was renal mass (n=230, 74.2%), with other less frequent conditions like non-functional kidney and upper tract urothelial carcinoma (UTUC) accounting for 28 (9%) and nine (2.9%), respectively.

**Table 1:** Sociodemographic and other parameters of participants (N=310) SD: Standard Deviation; BMI: Body Mass Index; UTUC: Upper Tract Urothelial Carcinoma

Demographics	Categories	Values
Gender, n (%)	Female	143 (46.1%)
	Male	167 (53.9%)
Age (Years)	Mean (±SD)	53.8 (±15.5)
	Range	19-92
Weight (Kg)	Mean (±SD)	79.7 (±16.4)
	Range	33-123
Height (m)	Mean (±SD)	1.6 (±0.1)
	Range	1.2-1.9
BMI (kg/m^2^)	Mean (±SD)	30.2 (±6.4)
	Range	11.5-57.5
Nephrectomy Type, n (%)	Radical	188 (60.6%)
	Partial	99 (31.9%)
	Nephroureterectomy	15 (4.8%)
	Simple/Total	7 (2.3%)
Side	Left	157 (50.6%)
	Right	150 (48.4%)
	Both	2 (0.6%)
Started Procedure Type, n (%)	Laparoscopy	172 (55.5%)
	Open	69 (22.3%)
	Robotic	69 (22.3%)
Indications, n (%)	Renal Mass	230 (74.2%)
	Non-Functional Kidney	28 (9.0%)
	UTUC	9 (2.9%)
	Renal Cyst	2 (0.6%)
	Renal Abscess	2 (0.6%)
	Pyelonephritis	2 (0.6%)
	Calculus	2 (0.6%)
	Other	34 (11.0%)

**Figure 1:**
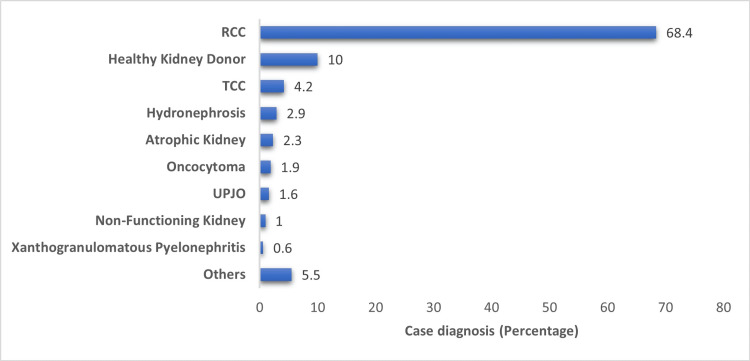
Different diagnosis among participants (N=310) RCC: Renal Cell Carcinoma; TCC: Transitional Cell Carcinoma; UPJO: Ureteropelvic Junction Obstruction

Figure 1 shows different diagnoses among participants (n=310, 100%). RCC is the most prevalent diagnosis, constituting 68.4% (n=212) of cases. Other notable diagnoses include healthy kidney donor at 31 (10%), transitional cell carcinoma (TCC) at 13 (4.2%), hydronephrosis at nine (2.9%), atrophic kidney at eight (2.3%), oncocytoma at six (1.9%), ureteropelvic junction obstruction at five (1.6%), and non-functioning kidney at three (1%). Xanthogranulomatous pyelonephritis appeared in two (0.6%) cases, while other less common conditions made up the remaining 5.5% (n=17) of the diagnoses.

Table 2 shows past medical conditions and comorbidities among participants. Notably, a majority reported no past renal history (n=232, 74.8%), while 74 (23.9%) had a past renal history including surgery. HTN was the most common comorbidity, affecting 198 (63.9%) participants. DM was present in 130 (41.9%), and dyslipidemia was reported in 65 (21.0%). Benign prostatic hyperplasia was confirmed in 67 (21.6%) cases. Coronary artery disease (CAD) and hypothyroidism were less frequent, affecting 50 (16.1%) and 14 (4.5%) patients, respectively. Other miscellaneous conditions were noted in 43 (13.9%) participants.

**Table 2:** Past medical conditions and comorbidities among participants (N=310)

Diseases	Presence	Frequency (Percentage)
Past Renal History	No	232 (74.8%)
	Yes (Including Past Renal Surgery)	74 (23.9%)
Benign Prostatic Hyperplasia	No	20 (6.5%)
	Yes	67 (21.6%)
Hypertension	No	112 (36.1%)
	Yes	198 (63.9%)
Diabetes Mellitus	No	158 (51.0%)
	Yes	130 (41.9%)
Dyslipidemia	No	218 (70.3%)
	Yes	65 (21.0%)
Coronary Artery Disease	No	229 (73.9%)
	Yes	50 (16.1%)
Hypothyroidism	No	266 (85.8%)
	Yes	14 (4.5%)
Other	No	236 (76.1%)
	Yes	43 (13.9%)

**Figure 2:**
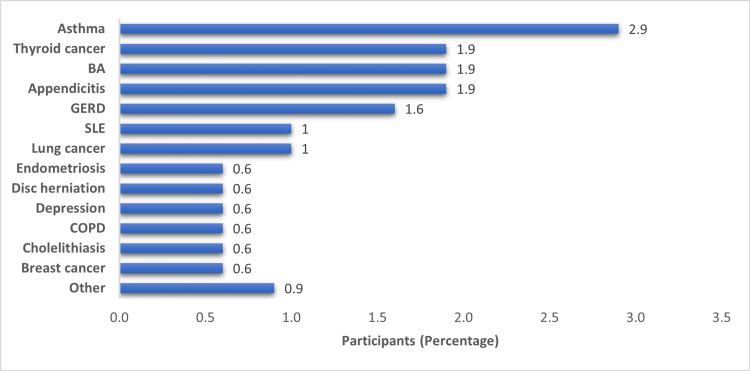
Other comorbidities among participants (n=43) BA: Biliary Atresia, GERD: Gastroesophageal Reflux Disease, SLE: Systemic Lupus Erythematosus, and COPD: Chronic Obstructive Pulmonary Disease

Figure 2 shows other comorbidities found among 43 participants in our study. The most common comorbidity was asthma, affecting nine (2.9%) of participants. Thyroid cancer, biliary atresia, and appendicitis each affected six (1.9%) patients of this subset. Gastroesophageal reflux disease (GERD) was present in five (1.6%) participants, followed by three (1%) participants each in systemic lupus erythematosus (SLE) and lung cancer. The remaining conditions were endometriosis, disc herniation, depression, chronic obstructive pulmonary disease (COPD), cholelithiasis, and breast cancer, reported in two (0.6%) participants each.

Table 3 shows different parameters before and after Interventions, Incidence of proteinuria, and rate of mortality. Notably, there was a decrease in estimated glomerular filtration rate (eGFR) from a pre-operative mean of 96.45 mL/minute (SD±19.352) to 78.41 mL/minute (SD±23.091) within one to three months postoperatively. The eGFR slightly improved to 80.02 mL/minute (SD±22.569) by 6-12 months postoperative. Serum creatinine levels increased from a preoperative mean of 72.75 µmol/L (SD±15.34) to 91.11 µmol/L (SD±25.48) in the first one to three months post surgery and slightly decreased to 88.40 µmol/L (SD±25.58) by 6-12 months. Blood urea nitrogen (BUN) levels showed a modest increase from preoperative values of 4.70 mmol/L (SD±1.58) to 5.59 mmol/L (SD±2.17) by 6-12 months post surgery. The mortality rate was low, with five (1.6%) participants passing away and the survivors having a mean survival time of 19.86 months (SD±18.417) postoperatively. Proteinuria was observed in 84 (27.1%) participants post surgery, with an average protein level of 89.94 mg/dL (SD±82.30) over an average period of 32.9 months (SD±24.9).

**Table 3:** Different parameters after interventions, incidence of proteinuria, and rate of mortality eGFR: estimated Glomerular Filtration Rate

Factors	Categories	Mean (± SD)	Min-Max (Range)
eGFR (ml/min)	Preoperative	96.45 (±19.352)	60-160
	Postoperative (1-3 Months)	78.41 (±23.091)	32-150
	Postoperative (6-12 Months)	80.02 (±22.569)	32-170
Serum Creatinine	Preoperative	72.75 (±15.34)	45-133.00
	Postoperative (1-3 Months)	91.11 (±25.48)	44-176.0
	Postoperative (6-12 Months)	88.40 (±25.58)	46-156.0
Blood Urea Nitrogen	Preoperative	4.70 (±1.58)	1.1-10.2
	Postoperative (1-3 Months)	5.64 (±2.175)	1.8-14.8
	Postoperative (6-12 Months)	5.59 (±2.170)	1.6-13.9
Deaths	No (n, %)	305	98.4%
	Yes (n, %)	5	1.6%
	Survival Months	19.86 (±18.417)	0.00-97.00
Post-Op Proteinuria	No (n, %)	226	72.9%
	Yes (n, %)	84	27.1%
	Level (mg/dl)	89.94 (±82.30)	30-500
	Period (Months)	32.9 (±24.9)	<1-111

Table 4 shows the differences in outcomes between radical and partial nephrectomy. Preoperatively, both nephrectomy types showed similar eGFR values with no significant difference (Radical: 97.15 mL/minute (SD±19.44); Partial: 97.59 mL/minute (SD±19.25) (p=0.856). However, significant differences emerged post-operatively. After one to three months, the eGFR was significantly lower in patients who underwent radical nephrectomy 73.09 mL/minute (SD±20.30) compared to those with partial nephrectomy 90.99 mL/minute (SD±24.13)) (p<0.001), a trend that persisted at 6-12 months (Radical: 74.36 mL/minute (SD±21.43); Partial: 92.63 mL/minute (SD±19.28) (p<0.001). Serum creatinine levels followed a similar pattern, with no significant difference preoperatively (Radical: 71.05 µmol/L (SD±15.13); Partial: 71.57 µmol/L (SD±17.65)) (p=0.794), but significant differences postoperatively at one to three months (Radical: 94.59 µmol/L (SD±24.94); Partial: 79.39 µmol/L (SD±23.00)) (p<0.001) and 6-12 months (Radical: 92.85 µmol/L (SD±26.46); Partial: 75.60 µmol/L (SD±22.38)) (p<0.001). BUN levels were not significantly different preoperatively but showed a significant difference at one to three months postoperative (Radical: 6.01 mmol/L (SD±2.35); Partial: 4.91 mmol/L (SD±1.62)) (p<0.001), though this difference was not maintained at 6-12 months (p=0.179). All mortality cases (n=4; 100%) post nephrectomy occurred in the radical nephrectomy group, with no deaths in the partial nephrectomy group. However, the survival months and rates of postoperative proteinuria did not significantly differ between the groups, except for the level of proteinuria, which was higher in the radical group 107.10 mg/dL (SD±98.68) compared to the partial group 62.80 mg/dL (SD±27.01) (p=0.031).

**Table 4:** Association, difference of parameters, and outcomes among two major types of nephrectomies ^(a)^ Chi-Square Test, ^(b)^ Fisher’s Test, and ^(c)^ Independent T-test eGFR: Estimated Glomerular Filtration Rate

Factors	Categories	Nephrectomy Type		Sig. Value
		Radical Mean (±SD)	Partial Mean (±SD)	
eGFR (ml/min)	Preoperative	97.15 (±19.44)	97.59 (±19.25)	0.856^c^
	Postoperative (1-3 Months)	73.09 (±20.30)	90.99 (±24.13)	<0.001^ c^
	Postoperative (6-12 Months)	74.36 (±21.43)	92.63 (±19.28)	<0.001^ c^
Serum Creatinine	Preoperative	71.05 (±15.13)	71.57 (±17.65)	0.794^ c^
	Postoperative (1-3 Months)	94.59 (±24.94)	79.39 (±23.00)	<0.001^ c^
	Postoperative (6-12 Months)	92.85 (±26.46)	75.60 (±22.38)	<0.001^ c^
Blood Urea Nitrogen	Preoperative	5.98 (±10.70)	5.36 (±7.02)	0.599^ c^
	Postoperative (1-3 Months)	6.01 (±2.35)	4.91 (±1.62)	<0.001^ c^
	Postoperative (6-12 Months)	6.16 (±6.22)	5.18 (±1.68)	0.179^ c^
Death	No (n, %)	185 (65.1%)	99 (34.9%)	0.302b^ b^
	Yes	4 (100.0%)	0 (0.0%)	
	Survival (Months)	20.77 (±18.93)	17.39 (±16.63)	0.139^ c^
1^st^ Time Postoperative Proteinuria	Negative	138 (65.1%)	74 (24.9%)	0.780 ^a^
	Positive	51 (67.1%)	25 (32.9%)	
	Level (mg/dl)	107.10 (±98.68)	62.80 (±27.01)	0.031^ c^
	Time from Procedure	32.59 (±26.75)	35.28 (±21.89)	0.664^ c^

Table 5 shows the association between various participant characteristics and mortality outcomes in participants. There were no significant differences in mortality based on gender, with 139 (97.2%) female and 166 (99.4%) male participants surviving. Age, weight, height, and BMI also showed no significant association with mortality, with p-values of 0.628, 0.924, 0.498, and 0.725, respectively. In terms of procedure type, survival rates were high across all methods: laparoscopy (97.7% survival), open surgery (98.6% survival), and robotic surgery (100% survival), with no significant differences noted. Pre-existing conditions such as past renal history, benign prostatic hyperplasia, HTN, DM, dyslipidemia, CAD, and hypothyroidism similarly showed no significant associations with mortality. Notably, the only significant association was observed with pre-operative eGFR, where participants who died had a significantly lower preoperative eGFR 73.80 mL/minute (SD±7.53) compared to survivors 96.83 mL/minute (SD±19.27) (p=0.008). Postoperative eGFR, serum creatinine, and BUN levels at various time points did not show significant differences between survivors and non-survivors. Levels of proteinuria and the time post-operation at which proteinuria was positive also did not differ significantly between the groups.

**Table 5:** Association of different parameters with mortality participants ^(a)^ Chi-Square Test, ^(b)^ Fisher’s Test, and ^(c)^ Independent T-test BMI: Body Mass Index; eGFR: estimated Glomerular Filtration Rate

Factors	Categories		Deaths/Mortality		Sig. Value
			No, n (%)	Yes, n (%)	
Gender		Female	139 (97.2%)	4 (2.8%)	0.185^b^
		Male	166 (99.4%)	1 (0.6%)	
Age		Mean (±SD)	53.80 (±15.62)	57.20 (±10.23)	0.628^ c^
Weight		Mean (±SD)	79.81 (±16.43)	79.10± (17.28)	0.924^ c^
Height (m)		Mean (±SD)	1.63 (±0.10)	1.60 (±0.08)	0.498^ c^
BMI (kg/m^2^)		Mean (±SD)	30.18 (±6.44)	31.20 (±7.48)	0.725^ c^
Procedure Type		Laparoscopy	168 (97.7%)	4 (2.3%)	0.828^ c^
		Open	68 (98.6%)	1 (1.4%)	
		Robotic	69 (100.0%)	0 (0.0%)	
Past Renal History (Renal Surgery)		No	229 (98.7%)	3 (1.3%)	0.598^ b^
		Yes	72 (97.3%)	2 (2.7%)	
Benign Prostatic Hyperplasia		No	20 (100.0%)	0 (0.0%)	1.000^ b^
		Yes	65 (97.0%)	2 (3.0%)	
Hypertension		No	111 (99.1%)	1 (0.9%)	0.657^ b^
		Yes	194 (98.0%)	4 (2.0%)	
Diabetes Mellitus		No	155 (98.1%)	3 (1.9%)	0.630^ b^
		Yes	129 (99.2%)	1 (0.8%)	
Dyslipidemia		No	214 (98.2%)	4 (1.8%)	0.577^ b^
		Yes	65 (100.0%)	0 (0.0%)	
Coronary Artery Disease		No	225 (98.3%)	4 (1.7%)	1.000^ b^
		Yes	50 (100.0%)	0 (0.0%)	
Hypothyroidism		No	262 (98.5%)	4 (1.5%)	1.000^ b^
		Yes	14 (100.0%)	0 (0.0%)	
Others		No	232 (98.3%)	4 (1.7%)	1.000^ b^
		Yes	43 (100.0%)	0 (0.0%)	
eGFR (ml/min)		Pre-Op	96.83 (±19.27)	73.80 (±7.53)	0.008^ c^
		Post-Op [1-3 Months]	78.51 (±23.30)	73.80 (±8.59)	0.653^ c^
		Post-Op [6-12 Months]	80.05 (±22.77)	78.60 (±8.79)	0.888^ c^
Serum Creatinine		Pre-Op	71.97 (±16.32)	81.00 (±14.20)	0.220^ c^
		Post-Op [1-3 Months]	90.82 (±26.05)	80.40 (±13.24)	0.374^ c^
		Post-Op [6-12 Months]	88.14 (±26.80)	79.20 (±11.23)	0.458^ c^
Blood Urea Nitrogen		Pre-Op	5.73 (±9.32)	5.12 (±0.77)	0.883^ c^
		Post-Op [1-3 Months]	5.65 (±2.19)	5.06 (±1.53)	0.548^ c^
		Post-Op [6-12 Months]	5.86 (±5.07)	4.36 (±1.14)	0.510^ c^
Proteinuria		Level (mg/dl)	89.82 (±82.81)	100.00 (-)	0.903^ c^
		Positive Protein Time	33.38 (±24.84)	24.75 (±28.74)	0.502^ c^

Table 6 shows the incidence of proteinuria among patients who underwent nephrectomy and are on angiotensin-converting enzyme (ACE) inhibitors postoperatively. Among those who had a radical nephrectomy (n=38, 100%), 16 (42.1%) tested positive for proteinuria, while 22 (57.9%) were negative for proteinuria. In comparison, for patients with partial nephrectomy, 18 (100%) had a slightly lower incidence, with seven (38.9%) testing positive and 11 (61.1%) testing negative. This data suggests a modestly higher incidence of proteinuria in patients who underwent radical nephrectomy compared to those who had partial nephrectomy, indicating a potential association between the type of nephrectomy and the likelihood of developing proteinuria among patients on ACE inhibitors.

**Table 6:** Incidence of proteinuria among ACE inhibitor users ACE: Angiotensin-Converting Enzyme

Nephrectomy Type	Negative, n (%)	Positive, n (%)	Total
Radical	22 (57.9%)	16 (42.1%)	38
Partial	11 (61.1%)	7 (38.9%)	18

## Discussion

Nephrectomy, performed as partial or radical, addresses various kidney conditions through laparoscopic, robotic, or open surgical methods. According to Kunath et al., partial nephrectomy and radical nephrectomy are the relevant surgical therapy options for localized RCC [11]. Many studies highlight the risk of proteinuria post nephrectomy [6,12]. However, comparative data on proteinuria outcomes between radical and partial nephrectomies are lacking. This study aimed to bridge this gap by examining proteinuria incidence from 2014 to 2022 in KAMC and analyzing the impact of each surgery type on kidney function. By assessing clinical data and risk factors associated with both surgical types, this study sought to inform patient care and healthcare policies, enhancing the understanding of nephrectomy’s benefits and risks.

Notably, the rate or incidence of RCC was found in 212 (68.4%) participants. This is consistent with global trends where RCC accounts for approximately 90% of renal malignancies [13]. Moreover, Wang et al. reported that RCC constitutes 2.4% of all global cancer diagnoses [14]. The high prevalence of RCC in our study underscores the need for continued research and intervention strategies focusing on this kidney cancer subtype.

The substantial proportion of healthy kidney donors (10%) in our study aligns with increasing global kidney donation trends, reflecting enhanced healthcare system capacities to facilitate living donor programs [15]. Representing other conditions like severe or chronic hydronephrosis due to obstruction or non-functioning kidney [16], and upper tract urothelial carcinoma [17]. Additionally, conditions like chronic pyelonephritis, renal tuberculosis, or severe calculi often necessitate nephrectomy [17], suggesting our cohort is representative of typical nephrectomy populations.

The comorbidity profile of our participants, with high prevalence rates of HTN (63.9%) and diabetes mellitus (41.9%), matches the broader chronic disease challenges in renal diseases. Clayman et al. demonstrated that new-onset diabetes predicts 10-year mortality more than HTN, with both conditions markedly increasing the risk of death post nephrectomy [18]. However, Charoensri et al. emphasized that partial nephrectomy is underutilized in patients with DM and HTN despite its association with better outcomes compared to radical nephrectomy [19]. These comorbidities are known to complicate post-nephrectomy surgical outcomes and may necessitate more nuanced perioperative care [20].

Our findings regarding the decrease in eGFR post-nephrectomy corroborate earlier studies reporting declines in kidney function as a common outcome following radical nephrectomies. Di Marco et al. revealed that 64% of patients experienced acute kidney injury and reduced renal function after radical nephrectomy [21]. Yan et al. further demonstrated that CKD is a common postoperative complication in patients undergoing radical nephrectomy for renal tumors [22]. Encouragingly, the partial recovery of eGFR at 6-12 months in our study aligns with evidence suggesting residual kidney compensation. Khanna et al. highlighted that partial nephrectomy causes less moderate renal function decline than radical nephrectomy, although severe dysfunction rates are similar [23]. Moreover, partial nephrectomy was associated with improved overall survival and reduced CKD risk compared to radical nephrectomy in RCC cases [24].

The mortality rate of 1.6% in our study is within the lower range reported in the literature, typically varying between 1% and 3% for nephrectomy procedures. Fontenil et al. reported 30-day mortality rates after nephrectomy ranging from 0.6% to 3.6%, influenced by age, disease stage, and surgery type [25]. Importantly, no deaths occurred in patients undergoing partial nephrectomy and robotic surgeries, supporting prior studies indicating lower complication rates with minimally invasive surgical techniques [26]. Similarly, Pyrgidis et al. demonstrated that radical nephrectomy significantly impacts renal function compared to partial nephrectomy, especially in those with preoperative poor renal function [27].

Our study found that proteinuria incidence (27.1%) was significantly higher in radical nephrectomy patients. This aligns with Krebs et al.’s findings that proteinuria post-nephrectomy may signal underlying renal damage and increased CKD risk [28].

Limitations and future research

While our study provides insightful findings, it is not without limitations. The retrospective design and the single-center nature may limit the generalizability of the results. Future multicenter studies could provide more generalized data and help validate our findings across different demographics and healthcare settings. Additionally, longitudinal studies could provide more insights into the long-term outcomes of nephrectomy patients, especially concerning CKD development and long-term survival.

Implications

Radical nephrectomy greatly reduces renal function, urging a cautious approach in surgical decision-making, especially for those with preexisting renal issues. It emphasizes the need for patient education about potential outcomes, advocating for nephron-sparing methods where possible. Healthcare systems should enhance postoperative care to prevent severe complications like end-stage renal disease. This necessitates further research into surgical techniques that conserve renal function and supportive postoperative medical therapies.

## Conclusions

Our study highlights the significant impact of radical nephrectomy on renal function, illustrating a compelling need for a tailored surgical approach, particularly for patients with compromised preoperative renal health. This study underscores the importance of considering nephron-sparing techniques as viable alternatives to mitigate the risks of severe renal impairment. Furthermore, it emphasizes the necessity for enhanced patient counseling and vigilant postoperative monitoring to manage and potentially prevent the progression to end-stage renal disease. Ultimately, these insights call for ongoing advancements in surgical practices and patient management strategies to optimize outcomes for individuals undergoing nephrectomy.
